# Congenital Transmission of Apicomplexan Parasites: A Review

**DOI:** 10.3389/fmicb.2021.751648

**Published:** 2021-09-29

**Authors:** Maura Rojas-Pirela, Lisvaneth Medina, Maria Verónica Rojas, Ana Isabel Liempi, Christian Castillo, Elizabeth Pérez-Pérez, Jesús Guerrero-Muñoz, Sebastian Araneda, Ulrike Kemmerling

**Affiliations:** ^1^Instituto de Ciencias Biomédicas, Facultad de Medicina, Universidad de Chile, Santiago, Chile; ^2^Instituto de Biología, Pontificia Universidad Católica de Valparaíso, Valparaíso, Chile; ^3^Facultad de Farmacia y Bioanálisis, Universidad de Los Andes, Mérida, Venezuela; ^4^Núcleo de Investigación Aplicada en Ciencias Veterinarias y Agronómicas, Facultad de Medicina Veterinaria y Agronomía, Universidad de Las Américas, Santiago, Chile; ^5^Facultad de Salud y Odontología, Universidad Diego Portales, Santiago, Chile

**Keywords:** Apicomplexa, congenital transmission, placenta, infection-immunology, host–parasite interactions

## Abstract

Apicomplexans are a group of pathogenic protists that cause various diseases in humans and animals that cause economic losses worldwide. These unicellular eukaryotes are characterized by having a complex life cycle and the ability to evade the immune system of their host organism. Infections caused by some of these parasites affect millions of pregnant women worldwide, leading to various adverse maternal and fetal/placental effects. Unfortunately, the exact pathogenesis of congenital apicomplexan diseases is far from being understood, including the mechanisms of how they cross the placental barrier. In this review, we highlight important aspects of the diseases caused by species of *Plasmodium, Babesia, Toxoplasma*, and *Neospora*, their infection during pregnancy, emphasizing the possible role played by the placenta in the host-pathogen interaction.

## Introduction

The phylum Apicomplexa constitutes a broad group of parasitic protists that include more than 6000 species (Votýpka et al., 2017), distributed in a wide diversity of environments, including soil, freshwater, and marine habitats (del Campo et al., 2019). Many of these parasites have significant clinical and economic relevance since they cause important human and veterinary diseases worldwide. In this context, malaria (*Plasmodium* species), babesiosis (*Babesia* sp.), toxoplasmosis (*Toxoplasma gondii*), neosporosis (*Neospora* sp.), and cryptosporidiosis (*Cryptosporidium parvum*) are diseases caused by apicomplexan parasites (Chakraborty et al., 2017; Martínez-Ocampo, 2018).

All those parasites present complex life cycles, infecting one or more hosts and undergoing multiple morphological and metabolic differentiation (Martínez-Ocampo, 2018; Jeninga et al., 2019). Additionally, in the life cycles of most of the apicomplexan, an alternation between asexual and sexual reproduction can be observed. Thus, in the different life cycles, three different states can be observed: sporozoite (infective stage), merozoite (a result of asexual reproduction), and gametocyte (germ cells) (Votýpka et al., 2017).

Apicomplexan parasites are characterized by having a complex of apical secretory organelles composed of micronemes, rhoptries, and dense granules. These organelles vary between species and are involved in host cell attachment, motility, invasion, and intracellular parasitophorous vacuole formation (Votýpka et al., 2017). In addition, all apicomplexan examined so far (except *Cryptosporidium* and *Gregarine*) possess an apicoplast (Salomaki and Kolisko, 2019), a relict plastid involved in several essential metabolic pathways, such as fatty acid, isoprenoid, and heme biosynthesis, that are crucial for parasite replication and establishment of infection (Ralph et al., 2004; Chakraborty, 2016).

Apicomplexan presents different transmission routes (Votýpka et al., 2017). However, for some species such as *Neospora*, *Babesia microti* (*B. microti*), and *Toxoplasma gondii (T. gondii)*, the infection from mother to fetus constitutes an important transmission route (Joseph et al., 2012; Hide, 2016; Khan et al., 2020). The incidence of congenital malaria, caused by *Plasmodium*, is relatively low in endemic regions. However, some reports have documented that the incidence can reach 37% (Ouédraogo et al., 2012; Olupot-Olupot et al., 2018). Regarding *T. gondii*, when primo-infection occurs during pregnancy, the congenital transmission rates are high (Hide, 2016), increasing from the first to the third trimester of pregnancy (McAuley, 2014). Importantly, these congenital infections can be detrimental to maternal and child health (Maldonado et al., 2017; Tegegne et al., 2019). The frequency of the transplacental transmission of these parasites is associated with different factors such as maternal and developing fetus immune systems and genetic background (Ortiz-Alegría et al., 2010; Ouédraogo et al., 2012; Gómez-Chávez et al., 2019), the passive transfer of maternal antibodies (Ortiz-Alegría et al., 2010; Dobbs and Dent, 2016) characteristics of the fetal environment (De Silva et al., 1982), and the functioning of the placenta as a physical barrier and immune organ (Robbins et al., 2012; Sharma and Shukla, 2017). Thus, the placenta is a crucial organ determining the probability of infection (Castillo et al., 2018).

The present review is focused on the congenital transmission of *Plasmodium, Babesia, T. gondii, Neospora* and the role of the placenta.

## Diseases Caused by Apicomplexan Parasites: Social and Economic Impact

Diseases caused by apicomplexan parasites affect millions of people, mainly in low- and middle-income countries located in the tropical and subtropical regions (Votýpka et al., 2017). Moreover, some of those infections have been included in the group of Neglected Tropical Diseases (NTD) (PLoS Neglected Tropical Disease, 2020), implying a significant social and economic impact that can reach millions of dollars each year (Laha et al., 2015; Ozawa et al., 2019; Stelzer et al., 2019).

**Malaria** is caused by five members of the genus *Plasmodium*, where *P. falciparum* and *P. vivax* species represent the most significant threat (Geleta and Ketema, 2016). Malaria is an important public health problem; it was estimated in 2018 that 228 million people were infected worldwide, and 405,000 malaria deaths were reported (WHO, 2019). Children aged under 5 years and pregnant women constitute the most vulnerable population (Gabrielli et al., 2016; WHO, 2019). The attempts to control and eradicate malaria present an estimated economic impact of 2.7 billion dollars (WHO, 2019).

**Babesiosis** is a globally distributed disease with malaria-like symptoms that affects elderly and immunocompromised patients (Kim et al., 2016), caused by erythrocytic parasites of the genus *Babesia.* Many of the 100 species of *Babesia* constitute a significant threat to humans, domestic animals, and livestock (Young et al., 2019), being in humans, *B. microti*, *B. divergens, B. duncani*, and *B. venatorum* the causal agents (Sun et al., 2014; Young et al., 2019). The mortality rate of this disease is approximately 5%; however, in case of infection through blood transfusion, the mortality rate increases to 19% (Katz and Dodd, 2019). In addition to human health effects, some *Babesia* species cause a significant loss to the cattle industry due to death, a loss of beef production of infected animals, and death (Mosqueda et al., 2012).

**Toxoplasmosis** is a zoonotic disease that affects approximately one-third of the world population (Ngô et al., 2017; Stelzer et al., 2019). The infection in immunocompetent individuals is generally asymptomatic; however, severe symptoms are observed in newborns (i.e., mental retardation, ocular disease) when the primoinfection occurs in the pregnant mother. Additionally, infection during pregnancy can cause abortion (Tegegne et al., 2016). Toxoplasmosis can be fatal in immunosuppressed patients since the reactivation of latent infection can lead to the development of encephalitis and, in some cases, reactivation of malignancies (Maciel et al., 2000; Wang Z.D. et al., 2017). In the case of animals, it is considered one of the leading causes of reproductive losses in small ruminants worldwide, which also play an important role in transmitting the parasite to humans. In some countries, such as Australia, the losses attributed to this disease can be up to 70 million dollars (Stelzer et al., 2019).

**Neosporosis** is a devastating worldwide disease responsible for abortions, neonatal mortality, and central nervous system diseases in animals. It is mainly caused by the species *Neospora caninum* (*N. caninum*) and *Neospora hughesi* (*N. hughesi*) and is transmitted by horizontal and vertical routes (Dubey, 2003; Donahoe et al., 2015). *N. caninum* affects dogs and cattle but occasionally infects horses, sheep, and deer, while *N*. *hughesi* only infects horses causing Equine Protozoal Myeloencephalitis (Dubey, 2003; Wobeser et al., 2009).

Neosporosis is associated with sporadic abortions (between 10 and 12.5%) in cattle herds with a frequent congenital transmission. However, the percentage of abortions increases (30–57%) in herds when the parasite is acquired by the pregnant mother (Dubey et al., 2007). *N. caninum* infections have a significant global economic impact; for instance, in New Zealand, the losses attributed to this disease exceed US$ 35.7 million (Reichel et al., 2013).

## Host–Apicomplexan Interactions at the Mammalian Placenta

As mentioned above, apicomplexan parasites can be transmitted congenitally. Within this group *Plasmodium* spp. and *T. gondii* are the most documented (Carlier et al., 2012). The placenta is a transitory organ that acts as the interface between the mother and fetus, which mediates nutrition and gas exchange between the fetus and the mother, ensuring growth and normal embryo-fetal development and supporting the maternal changes associated with pregnancy (Griffiths and Campbell, 2015; Castillo et al., 2018). The chorioallantoic is the main placenta in mammals during middle to late gestation and develops from the endometrium of the uterus and the trophoblast of the embryo. According to the extent of trophoblast invasion into the uterus, placentation is classified into hemochorial (highly invasive), endotheliochorial (moderate invasive) or epitheliochorial (low invasive) (Liempi et al., 2020). In the case of the human placenta, it is classified as discoidal, villous, and hemochorial, made up of a fetal portion that (originates from the *Chorion frondosum*) and a maternal portion or decidua basalis (originates from the endometrium) (Castillo et al., 2018).

The functional units in the human placenta are the chorionic villi, formed by the trophoblast a lining epithelium formed by two types of cell populations, undifferentiated cytotrophoblasts (CT) and fully differentiated syncythiotrophoblasts (ST), and the villous stroma (VS) (Gude et al., 2004; Castillo et al., 2018). The trophoblast is connected to and separated from the villous stroma (VS), the fetal connective tissue by a basal lamina, a specialized structure of extracellular matrix (ECM) (Benirschke et al., 2012). Therefore, trophoblast, basal *laminae*, and VS, the latter containing fetal capillaries, form the placental barrier that pathogens must cross to infect the fetus during transplacental transmission (Kemmerling et al., 2019). However, the placenta can protect the developing fetus from some kind of pathogens (Zeldovich et al., 2013). That is the case of the kinetoplastid *Trypanosoma cruzi* (*T. cruzi*), where only a few parasite antigens and DNA can be identified in human chorionic villi (Duaso et al., 2010). Additionally, the presence of *T. cruzi* activates local placental defense mechanisms such as the epithelial turnover of the BLT and secretion of pro-inflammatory cytokines through Toll-like receptors (TLR) activation and NFkB signaling (Liempi et al., 2016, 2019; Castillo et al., 2017a, b).

The ovine and bovine placenta are of cotyledonary shape, villous and epitheliochorial. The fetal component is formed by the fusion of the avascular chorion and the vascular allantois. The placental barrier is composed by six tissue layers: maternal capillary endothelium, maternal endometrial connective tissue, maternal endometrial epithelium, trophoblast, chorionic connective tissue, and fetal endothelium (Benirschke et al., 2012; Liempi et al., 2020).

The type of placental barrier has been associated with the probability of transmission of pathogens. Thus, it has been proposed that in the hemochorial placenta, where the trophoblast is in direct contact with maternal blood, the placental infection and, therefore, the transmission to the fetus is facilitated. Moreover, considering that the complexity of the placental barriers increases from hemochorial (human) to epitheliochorial (ovine), it could be assumed that the parasites cross the hemochorial barrier more easily. However, the same hemochorial barrier favors the transfer of maternal antibodies to the fetus, and in less invasive placentas, a greater variety of pathogens is observed (Capellini et al., 2015; Liempi et al., 2020).

Unfortunately, most pathogens, including apicomplexan, can surpass the placental barrier and infect the fetus. As we will see in the following sections, these parasites use very similar adhesion mechanisms to the placenta, based on the modulation of the expression of adhesion molecules on the surface of placental cells. Additionally, the presence of these pathogens induces alterations in the immune response and a change in the Th1/Th2 balance, favoring the activation of defense mechanisms based on cellular immunity, which harms placental function and fetal growth.

## Congenital Malaria

**FIGURE 1:**
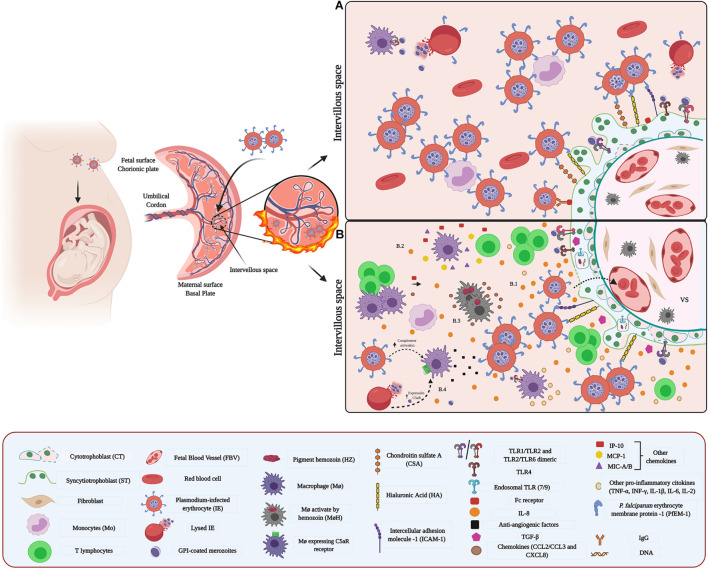
Host–pathogen interaction between *Plasmodium* and placenta: **(A)** Adhesion and sequestration of parasite-IEs. IEs express parasite-derived proteins on their surface. For example, the PfEMP1 protein family is highly expressed in placental IEs and acts as a surface antigen and ligand for their adhesion and sequestration. PfEMP1 selectively binds to specific placental receptors such as CSA. Other receptor molecules such as HI and ICAM-1, expressed in ST, participate as key molecules in the adhesion process. **(B)** Microenvironment and inflammatory response **(B.1,B.2)** Inflammation in the IVS is attributed to chemokines and cytokines secreted by maternal Mo, T cells, and ST. **(B.3)** Parasite’s hemozoin induces activation of MΦ, and subsequent release of chemokines. **(B.4)** Schizonts activate C5 and rupture releasing parasite components containing GPI that induce expression of C5aR and activate MΦ.

Malaria represents a high risk to the pregnant woman, fetus, and newborn. In some endemic areas, the prevalence of exposure to infection during pregnancy is 35%. Globally, malaria causes each year over 10,000 maternal and 75.000–200,000 neonatal deaths (Dombrowski et al., 2018; WHO, 2019). Congenital malaria is characterized by the sequestration of erythrocytes infected with *Plasmodium* parasites and the infiltration of immune cells within the intervillous space (IVS) of the placenta (Figure 1A), and the acquisition of a dark color of the basal plate, the fetal membranes (FM), fetal capillaries (FC), and monocytes (Mo) in the IVS, due to the deposition of the hemozoin malarial pigment (Parekh et al., 2010; Sharma and Shukla, 2017). The presence of parasites causes a pro-inflammatory environment in the placenta, leading to structural and functional alterations (Figure 1B). Placental damage, in turn, alters the nutrient exchange system between mother and fetus, leading to pregnancy-related complications including abortion, stillbirth, intrauterine growth retardation (IUGR), and low birth weight (LBW) (Briand et al., 2016; Sharma and Shukla, 2017). It has been proposed that due to hormonal and immunological changes in pregnancy (McLean et al., 2015), together with the fact that the parasite is localized inside red blood cells, it can more easily evade the maternal immune response (Sharma and Shukla, 2017). Interestingly, first-time pregnant women are more susceptible to malaria infection than women conceived a second or third time. This resistance to malaria infection in multigravida women is attributed to the development of placental parasite-specific immunity (Soulard et al., 2011).

Any of the Plasmodium species can cause congenital malaria; however, depending on the geographic region, infection by a specific species may predominate. Thus, in endemic regions of the Indian and African subcontinent, most congenital malaria cases are due to *P. falciparum* and *P. vivax* (Bhatia et al., 2016), while in European countries, *P. malariae* and *P. vivax* are predominate (Vottier et al., 2008). In South America, *P. vivax* accounts for the majority of malaria cases (Recht et al., 2017). In addition, *P. ovale* is a rare causative agent of congenital malaria (Jenkins, 1957; Penazzato et al., 2007), is limited to specific areas of tropical Africa and islands in the Western Pacific (Collins and Jeffery, 2005).

### Adhesion of *Plasmodium* to the Placenta

Plasmodium parasites invade red blood cells and induce the expression of parasite-derived proteins on the cell surface of the infected erythrocyte (IEs) (Chan et al., 2014). These parasite-derived proteins, known as variant surface antigens (VSAs), enable the IEs to adhere to the endothelium and subsequently infect different organs, including the placenta (Autino et al., 2012). In addition, VSAs bind to serum proteins forming red blood cell aggregates (rosetting), allowing the evasion of the host immune response, and establish chronic infection (Wahlgren et al., 2017). Among, VSAs *P. falciparu*m erythrocyte membrane protein 1 (PfEMP1) is a major surface antigen involved in vascular adhesion and sequestration of IEs (Chan et al., 2014). Notably, fEMP1 proteins are encoded by a polymorphic *var* multigene family (approximately 60 copies per genome), characterized by expression through allelic exclusion, allowing switching between PfEMP1 proteins and subsequent modification of the antigenic and binding properties of IEs (Wahlgren et al., 2017).

PfEMP1 proteins mediate the adhesion of IEs to different receptors and surface molecules including integrins (Chesnokov et al., 2018), endothelial protein C receptor (EPCR) (Gillrie et al., 2015), CD36 (Hsieh et al., 2016), intercellular adhesion molecule-1 and 2 (ICAM-1/2) (Lennartz et al., 2019), proteoglycans, such as chondroitin sulphate A (CSA) (Pehrson et al., 2016), glycosaminoglycans (GAGs), such as hyaluronic acid (HA) (Beeson and Brown, 2004), *P*-selectin, *E*-Selectin (Metwally et al., 2017) and Platelet/endothelial cell adhesion molecule-1 (PECAM-1) (Berger et al., 2013). In the placenta, the PfEMP1 variant VAR2CSA is predominantly expressed in IEs (Tuikue Ndam et al., 2005) and is the main VSA responsible for parasite-binding tropism (Nunes and Scherf, 2007; Smith, 2014) in this organ (Benavente et al., 2018). Notably, a selective accumulation of mature asexual stages of *P. falciparum*- IEs occurs in the IVS and chorionic villi surface (Beeson et al., 2002; Muthusamy et al., 2004; Figure 1A). The accumulation of IEs is mediated by receptors and molecules on the ST surface, macrophages (MΦ) monocytes (Mo), the fibrinoid deposits, and blood vessels in the VS (Beeson et al., 1999; Matejevic et al., 2001; Sugiyama et al., 2001).

Chondroitin sulphate A is the principal placental IEs receptor (Fried et al., 2006; Chishti, 2015). However, other molecules such as HA, non-immune immunoglobulins (IgG/IgM), and ICAM-1, present on the ST, can act as adhesion receptors (Maubert et al., 1997; Sartelet et al., 2000; Flick et al., 2001; Beeson and Brown, 2004; Barfod et al., 2011; Figure 1A). CD38 is only localized in the cytoplasm of stromal cells, fibroblasts, and MΦ, where the presence of IEs might influence its expression without being directly implicated in the sequestration of these (Sartelet et al., 2000). On the other side, HA is associated mainly with the aggregation or clumping of IEs (Beeson and Brown, 2004). Importantly, the expression of the different receptors is influenced by *P. falciparum-*induced proinflammatory cytokines (Vásquez et al., 2013).

In addition, other factors determine the accumulation and the sequestration of IEs in the placenta, including the low placental blood flow associated with trophoblast conformational changes, for the formation of cytotrophoblastic prolongations, known as “Coan-Burton bridges” (Moraes et al., 2013), IEs cell deformability (Safeukui et al., 2018), and the presence of Mo (Sugiyama et al., 2001). The adhesion and invasion of IEs also depend on the type of *Plasmodium* species. Thus, *P. vivax* and *P. falciparum* can bind to placental CSA and HA. However, *P. vivax* cannot bind to CD36 and ICAM-1 (Chotivanich et al., 2012), ligands widely used by *P. falciparum*.

So far, the *Plasmodium* mechanisms of adhesion to the placenta are the best known among apicomplexan parasites. However, as we will see in the following sections, these mechanisms are preserved in related apicomplexan parasites such *Babesia*, *Toxoplasma*, and *Neospora*. In these organisms, the parasite proteins expressed in the infected cells, or the proteins present on the parasite’s surface play fundamental roles in the adhesion and infection processes in the placenta.

### Immune Response Against *Plasmodium*

The development of severe malaria during pregnancy depends on parasite and host factors, including the immune response (Clark, 2019). During pregnancy, an increase of hormones, including cortisol, progesterone, estradiol, and testosterone, polarize the immune response toward a Th2-type one, characterized by an increase of regulatory T cells (Tregs) and anti-inflammatory cytokines (Robinson and Klein, 2012). The presence of the parasite alters this response causing infiltration of immune cells and a strong inflammatory response (Suguitan et al., 2003; Chêne et al., 2014).

Toll-like receptors, particularly TLRs-2 -4 -7-and -9, expressed on the trophoblast and cells of the VS, play a key role in the immune response against *Plasmodium* (Lucchi et al., 2011; Zhu et al., 2011; Barboza et al., 2014, 2017; Reis et al., 2020). TLR- 2 and -4 recognize glycosylphosphatidylinositol (GPI) anchors (Krishnegowda et al., 2005), while nucleic acids and hemozoin are recognized by TLR7 and TLR9 (Coban et al., 2005; Parroche et al., 2007; Figure 1B). The expression of TLR-4 and -9 is regulated by *Plasmodium* (Barboza et al., 2017) and polymorphisms of the TLRs have been associated with the susceptibility to infection (Hamann et al., 2010).

The binding of IEs and parasite antigens to TLR expressed on the trophoblast promotes the activation of MAPK pathways leading to the secretion of pro-inflammatory cytokines, including TNF-α, IFN-γ, TGF-β, and IL-8, and consequent activation of immune cell (Lucchi et al., 2008; Reis et al., 2020; Figure 1B). Concomitantly, the chemokines interferon γ–induced protein-10 (IP-10), monocyte chemoattractant protein-1 (MCP-1), and macrophage inflammatory protein (MIP)-1a/b expressed in maternal white blood cells (WBCs) contribute to the accumulation of Mo and MΦ in the IVS (Suguitan et al., 2003). Then, the presence of the immune cells and cyto/chemokines leads to a Th1-type immune response, where in addition to the cytokines mentioned above, IL1β, IL-2, IL-6, (Dobaño et al., 2018) and IL-8 (Fried et al., 1998) are secreted (Figure 1B). Thus, T cells proliferation is induced, and MΦ phagocytosis is enhanced, aiming to limit the parasite’s replication (Ozarslan et al., 2019).

Overproduction of cytokine IL-10, an immunoregulating cytokine, is associated with response to placental malaria infections (Suguitan et al., 2003; Chêne et al., 2014) attenuating the detrimental effects on the placental barrier exerted by the exacerbated inflammatory response (Okamgba et al., 2018). Alternatively, the increase in IL-10 could modulate lymphoid dendritic cell (DC) subpopulations shifting the Th1/Th2 balance toward a TH1 response and consequently promote infection (Diallo et al., 2008).

Mo-secreted IL-1β in response to IEs alters the amino acid transport system in the trophoblast impairing their transfer to the fetus and contributes to PM-associated FGR’s pathogenesis. Moreover, the accumulation of Mo in the malarial placenta promotes the decrease of insulin-like growth factor-1, one of the most influential factors in fetal growth and life (Umbers et al., 2011).

Different MΦ populations are related to functional modification of the placenta and maternal-fetal tolerance (Ordi et al., 1998; Gaw et al., 2019). Thus, maternal-intervillous monocytes (MIM) are associated with massive chronic villositis and placental damage (Ordi et al., 1998). Likewise, a decrease in the anti-inflammatory M2-type Hofbauer cells (HBC), located in the VS, is associated with LBW in placental malaria (Gaw et al., 2019).

*Plasmodium* also activates the complement cascade (C) and subsequently modulates the release of the anti-angiogenic factors (Conroy et al., 2011; Chau et al., 2017; Figure 1B). Together, an excessive mononuclear cells activation, such as MΦ, and an increase of soluble vascular endothelial growth factor (VEGF) receptor-1 (sFlt-1), which reduce the bioavailability of the pro-angiogenic VEGF and placental growth factor (PGF), impair normal angiogenesis (Azimi-Nezhad, 2014; Chau et al., 2017) and trigger functional placental insufficiency, and ultimately, FGR (Conroy et al., 2011).

The presence of Hemozoin (Hz) is another determining factor in the placental malaria immune environment. Hz can stimulate the trophoblast, MΦ, and DCs to secrete chemokines and cytokines [CCL2, CCL3, and CXCL8 and tumor necrosis factor (TNF)], contributing to the recruitment of peripheral blood mononuclear cells, especially Mo (Lucchi et al., 2011) and inflammatory response (Moormann et al., 1999; Abrams et al., 2003; Figure 1B). In addition, phagocytosis of this pigment increases the expression of matrix metalloproteinase (MMPs), particularly MMP-9, in the trophoblast (Clark, 2019) inducing cleavage and liberation of the extracellular domain of Syndecan-1; therefore allowing the adherence of IEs and promote their accumulation in the placenta (Clark, 2019).

## Congenital Babesiosis

**FIGURE 2:**
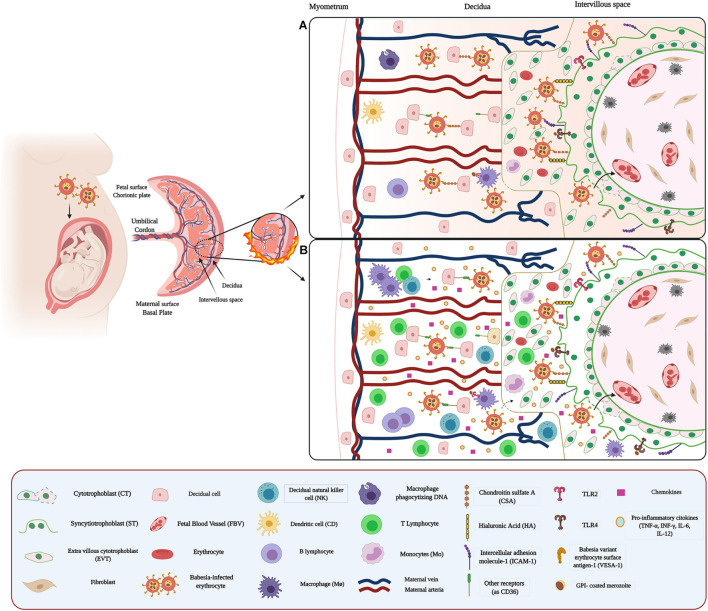
Host–pathogen interaction between *Babesia* and placenta: **(A)** Adhesion and sequestration of *Babesia*-IEs Infection of erythrocytes by the parasite induces expression of parasite-derived proteins on the surface of the IEs. Babesia-infected IEs highly express the VESA1 family and possibly act as a surface antigen and ligand for adhesion and sequestration of IEs in the placenta. VESA1 selectively binds to specific placental receptors, such as CSA, HA, and ICAM-1, expressed by ST, participate as key molecules in this adhesion process. In addition, some receptors such as CD36 could also facilitate the adhesion of IEs in other regions of the placenta, such as the decidua. **(B)** Microenvironment and inflammatory response against *Babesia*. Inflammation in the IVS and decidua could be attributed to chemokines and cytokines secreted by maternal MΦ, Mo, B cell, and T cells, as well as ST.

Congenital babesiosis, caused by *B. microti* and *Babesia* sp., is considered a rare disease; to date, a total of 10 cases have been reported in the United States (Esernio-Jenssen et al., 1987; New et al., 1997; Sethi et al., 2009; Aderinboye and Syed, 2010; Cornett et al., 2012; Joseph et al., 2012; Yager et al., 2014; Surra and Jesus, 2015; Saetre et al., 2018). Although transplacental babesiosis has been reported, so far, no histological studies have been conducted in *Babesia*-infected placentas. However, it has been proposed that *B. microti* sporozoites can invade red blood cells and cross the placental barrier and infect the fetus (Figure 2A). On the other side, the presence of the parasite in amniotic fluid has been demonstrated (Cornett et al., 2012). During gestational babesiosis, clinical manifestations similar to the HELLP syndrome (hemolysis, elevated liver enzymes, and low platelets) may develop, causing significant maternal and neonatal morbidity and mortality (Khangura et al., 2019). In infants, in addition to presenting hemolytic anemia, thrombocytopenia, and fever, hepatosplenomegaly is one of the most common symptoms (Saetre et al., 2018).

Babesiosis has also been documented in other mammals such as canines, felines, cattle, and rodents (Schoeman, 2009; Tołkacz et al., 2017). In these animals, babesiosis is caused by several species of babesia, which are relatively host-specific. However, all *Babesia* species can be transferred to other hosts through infected blood (Cox, 1982). Interestingly, in rodents, the placental transmission of *B. microti* is higher than in other mammals, including humans (Tołkacz et al., 2017; Tufts and Diuk-Wasser, 2018).

### Adhesion of *Babesia* to the Placenta

The pathogenesis of Babesia is strikingly similar to that of malaria (Figure 2A). Thus, alterations in the adhesive properties of IEs due to antigenic modifications of their membrane (Cooke et al., 2005; Hutchings et al., 2007), caused by the expression of a variant of the parasite-derived erythrocyte surface antigen protein (VESA), can be observed (Jackson et al., 2014). VESAs are proteins encoded by various families of ves1α and ves1β genes and secreted by the parasite (Allred et al., 2000; Allred, 2019). In addition, VESAs appear to play essential roles in pathogenicity (Pedroni et al., 2013), immune evasion (Jackson et al., 2014), persistence, and survival of the parasites (Allred, 2019). VESAs, variant erythrocyte surface antigenic- 1 (VESA1) protein, is one of the most studied in *B. bovis*. This protein acts as an endothelial cell ligand and mediator of antigenic variation in *B. bovis*-IEs (O’Connor and Allred, 2000; Wang et al., 2012). The host cell adhesion receptors for VESA have not yet been identified (Gallego-Lopez et al., 2019). However, structural similarities between fEMP1 and VESA1 suggest that the latter could bind to equivalent host cell receptors of PfEMP1 such as CD36 (Hutchings et al., 2007), ICAM-1, *P*-selectin, CSA, and CD31 (Gallego-Lopez et al., 2019; Figure 2A). Furthermore, studies performed with *B. bigemina* and *B. ridhaini* have demonstrated the adhesion IEs to thrombospondin (PST) (Parrodi et al., 1990) present in endothelial cells (Baruch et al., 1996).

Importantly, the severity of babesiosis is highly dependent on the babesia species (Cooke et al., 2005). For example, babesiosis caused by *Babesia microti, B. bovis, B. Lengua*, and *B. canis* is associated with the development of severe syndromes such as brain babesiosis and multi-organ failure due to the sequestration of IEs in the organ’s microvasculature (Wright et al., 1979; Aikawa et al., 1992; Bosman et al., 2013; Sondgeroth et al., 2013; Ripoll et al., 2018; Schetters, 2019).

### Immune Response Against *Babesia*

There are few reports related to the placental immune response against *Babesia*. However, it has been suggested that pro-inflammatory cytokine activation and erythrocyte adhesion in babesiosis are similar to malaria (Krause et al., 2007; Figure 2B). Some studies have even documented that prior infection with *B. microti* protects against fatal malaria in mice and primates through a mechanism of cross-protection (van Duivenvoorde et al., 2010; Efstratiou et al., 2020).

Toll-like receptors also recognize *Babesia* through the presence of its surface of GPI-linked molecules. The main TLRs studied regarding *Babesia* species are TLR-3 and -4, which can signal downstream through MyD88 independent pathways (Skariah et al., 2017). Similarly, TLR2 could play a role in this inflammatory response, mediating MΦ activation in the placenta. Previous studies have shown that lipid extracts from *B. bovis* stimulate TLR-2-mediated MΦ activation (Gimenez et al., 2010; Figure 2B) and consequently induces the secretion of pro-inflammatory cytokines, including TNF-α, IL-1, IL-12, and the immunomodulating IL-8 and nitric oxide (NO) (Shoda et al., 2000; Terkawi et al., 2015). In addition, T lymphocytes and natural killer (NK) cells, responsible for INF-γ production, and B lymphocytes are essential elements in the immune response against this parasite (Krause et al., 2007; Vannier and Krause, 2012; Yi et al., 2018; Figure 2B). Notably, the DNA of this parasite also has immunomodulatory effects on both B cells and MΦ, through the induction of IL-12, TNF-α, and NO production (Shoda et al., 2001).

*Babesia*, like *Plasmodium*, increases in the expression of MCP-1 and MIP-1a chemokines, contributing to the accumulation of Mo and MΦ in the IVS (Skariah et al., 2017; Zhao et al., 2020). Also, other chemokines such as CCL4 (MIP-1β) and CCL5 (RANTEs) have been associated with an inflammatory process in response to this parasite (Skariah et al., 2017).

## Congenital Toxoplasmosis

Congenital toxoplasmosis is one of the leading causes of infant morbidity and mortality (Melamed et al., 2010). Although it is a disease with global distribution, its prevalence is markedly variable from one region to another (Melamed et al., 2010; Torgerson and Mastroiacovo, 2013), with a global estimated incidence of 190100 annual cases (Torgerson and Mastroiacovo, 2013). Congenital toxoplasmosis occurs predominantly after primary infection in pregnant women (Pappas et al., 2009). However, congenital transmission has been reported in pregnant women infected before pregnancy (Pons et al., 1995; Vogel et al., 1996), chronically infected women, in whom the *T. gondii* infection was reactivated due to immunosuppression by HIV or by drug treatment for underlying diseases (Marty et al., 1994; Bachmeyer et al., 2006), and in women previously infected with one serotype who developed a new infection with a second serotype acquired during pregnancy (Fortier et al., 1991; Valdès et al., 2011).

**FIGURE 3:**
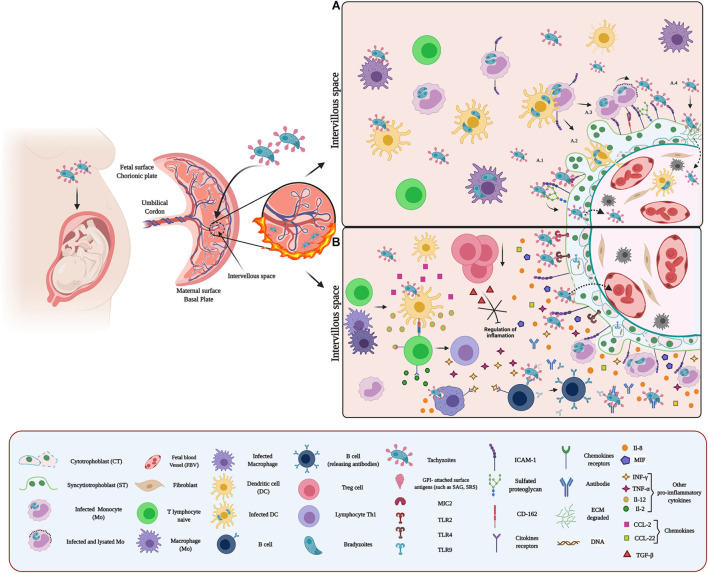
Host–pathogen interaction between *Toxoplasma* and placenta: **(A)** Adhesion of *T. gondii* in the placenta. *Toxoplasma* adhesion to the placenta can occur through proteins belonging to the SAG, SRS, and MIC families. These proteins expressed on the surface of the parasite act as surface antigens and ligands for molecules present on the surface of placental cells. These surface antigens could bind to GAGs, glycoproteins, adhesion molecules, and some extracellular matrix components. Once attached to the placenta, *T. gondii* crosses the placenta by different mechanisms: (i) without altering the integrity of the barrier **(A.1)**; (ii) infecting immune cells (such as CDs) and using them as Trojan horses **(A.2)**; (iii) exploiting the natural routes of leukocytes extravasation and coordinating its exit with the arrival at the target organ, thus minimizing attack by the immune system during the vehicle transition to target cells **(A.3)**; and (iv) and promoting EMC degradation, through modulation of MMP expression and secretion **(A.4)**. **(B)** Microenvironment and inflammatory response against *T. gondii*. Recognition of *T. gondii* by TLR (TLR2/4/9) induces IL-8 expression and secretion. Additionally, infection by this parasite promotes the expression of MIF and CCL-2. Recognition of the parasite by immune cells (such as CDs and MΦ) activates an inflammatory response characterized by cytokine and chemokine production such as IL-12, CCL-2, and CCL-22, which activate naive T cells. Subsequently, these cells differentiate to Th1 and produce cytokines (such as IL-2, INF-γ, and TNF-α) that promote an exacerbated pro-inflammatory environment, which stimulates the recruitment and activation of other immune cells at the site of infection. Additionally, infection by *T. gondii* promotes a decrease in Tregs, contributing to the exacerbated inflammatory reaction.

During congenital transmission, the parasite crosses the placenta directly or with the help of infected immune cells, Mo, and DCs, using them as “Trojan horses” (Figure 3A; Robert-Gangneux et al., 2011; Arranz-Solís et al., 2021), inducing phenotypic and functional alterations in infected cells, including overexpression of adhesion molecules and receptors, hypermotility, and downregulation of cytokine expression (Lachenmaier et al., 2011; Masocha and Kristensson, 2012; Sanecka and Frickel, 2012; Arranz-Solís et al., 2021). The parasite induces an inflammatory response (Figure 3B) that compromises mother and child health and the success of pregnancy (Robert-Gangneux et al., 2011). In addition, *T. gondii* causes severe neuro-ocular alterations such as chorioretinitis and the worst-case fetal death (Melamed et al., 2010). The severity of congenital Toxoplasmosis is inversely related to the gestational age at the time of primary maternal infection, implying that maternal infections in the first trimester of pregnancy lead to more serious clinical manifestations (McAuley, 2014). Besides, the clinical outcome of congenital Toxoplasmosis also is related to the *T. gondii* genotypes (Gilbert et al., 2008; McAuley, 2014), being the type I and II isolates related to more severe congenital Toxoplasmosis (Fuentes et al., 2001; Hutson et al., 2015; Rico-Torres et al., 2016; Lahmar et al., 2020; Arranz-Solís et al., 2021).

*Toxoplasma gondii* infects warm-blooded animals, including birds, cats, sheep, dogs, cows, goats, and pigs (Mondragon et al., 1998; Dubey et al., 2003; Stelzer et al., 2019); being isolates type II III the most frequent genotypes (Lachkhem et al., 2021). In some animals, the congenital transmission frequency is also high (Duncanson et al., 2001; Dos Santos et al., 2016) and is associated with fetal loss and economic losses (Stelzer et al., 2019). Interestingly, the potential risk of parasite transmission in animals through ingestion of contaminated milk has been suggested (Dubey et al., 2014; Ossani et al., 2017), increasing, therefore, the risk of zoonotic infection.

### Adhesion of *Toxoplasma gondii* to the Placenta

*Toxoplasma gondii* cell invasion is a multi-step process that depends on the parasite’s motility, surface antigens, and the sequential secretion of proteins present in the apical secretory organelles (Carruthers and Boothroyd, 2007; Robert-Gangneux and Dardé, 2012).

The invasion process starts with low-affinity interaction between *T. gondii* surface molecules, including glycosylphosphatidylinositol (GPI)-attached surface antigens (SAG), SAG-related sequence proteins (SRS), and non-SAG-related surface antigens (SUSA) and the cell membrane of the host cell (Pollard et al., 2008; Blader and Saeij, 2009). The SAGs multigenic family comprises more than 150 genes distributed and tandemly arrayed throughout the genome (Lekutis et al., 2001; Jung et al., 2004). SAG and SRS1 proteins are predominantly expressed on the tachyzoite (rapidly dividing parasites) surface cells (Tomavo, 1996; Manger et al., 1998). Regarding the SUSA molecules, 31 genes have been identified, and the proteins are highly expressed in bradyzoites (slow dividing parasites) (Pollard et al., 2008). Significantly, all these surface antigens are also involved in immune modulation, virulence, and protection of the parasite to survive in the environment (Lekutis et al., 2001; Crawford et al., 2009), for what they are considered redundant systems of adhesion to the host cell (Manger et al., 1998). The host cell surface molecules include GAGs, proteoglycans, and glycosaminoglycan-like carbohydrates (Jacquet et al., 2001; He et al., 2002; Azzouz et al., 2013). In addition, proteins of apical secretory organelles also play a key role in parasite-cell adhesion. For example, rhoptry proteins (ROP 2, ROP 4) and dense granules protein (GRA2) are lectins that bind to sulfated GAGs and then participate in the moving junction complex (MJ complex) (Azzouz et al., 2013).

Additionally, *T. gondii* is recognized and adheres to ICAM-1 on the host cell surface. Thus, micronemal proteins (MICs), such as MIC2, binds to ICAM-1 and facilitate the transmigration of the parasite through the blood-brain barrier (Lachenmaier et al., 2011). Also, interactions with ECM laminin (Furtado et al., 1992) and sialylated oligosaccharides (Blumenschein et al., 2007) have been reported. In the placenta, *T. gondii* infects different cell types, including decidual cells, extravillous trophoblasts (EVT), and the trophoblast (Abbasi et al., 2003; Robbins et al., 2012). The parasite’s binding to these cells is mediated by sulfated proteoglycan and ICAM-1 (Abbasi et al., 2003; Barragan et al., 2005; Figure 3A.1). ICAM-1 is abundantly expressed at intercellular junctions and the surface of in human trophoblast cell line (BeWo) (Barragan et al., 2005). Also, *T. gondii* induces syncytial expression of this adhesion molecule (Juliano et al., 2006) increasing the adhesion of *T. gondii*-infected immune cells to the trophoblast (Pfaff et al., 2005). Thus, parasite infection of Mo and maternal leukocytes promotes their adhesion to trophoblasts through the upregulation of ICAM-1, contributing to placental and ultimately fetal infection (Xiao et al., 1997; Pfaff et al., 2005). Moreover, decidual DCs could serve as a vehicle for *T. gondii* to travel across the placenta and infect the fetus (Sanecka and Frickel, 2012; Arranz-Solís et al., 2021; Figure 3A.2). In addition, *T. gondii* can exploit natural routes for leukocyte extravasation to cross the placental barrier (Figure 3A.3). In vascular endothelium, CD162 is recognized by the vascular adhesion molecule-1 (VCAM-1) and E-selectin of infected cells mediating their attachment (Harker et al., 2013). Interestingly, CD162 has been identified in trophoblast cell lines (Multhaup et al., 2018).

The adhesion of infected immune cells to the endothelium serves as a signal to the parasite, allowing to schedule its leukocyte output at the target organ and minimizing the attack by the immune system (Baba et al., 2017). Thus *T. gondii* reaches the placenta inside maternal leukocytes and adheres to the trophoblast (Figure 3A.3). In the case of Mo, the presence *T. gondii* antigens, such as soluble tachyzoite antigen (STAg), induces the overexpression of macrophage migration inhibitory factor (MIF), allowing the adhesion to the trophoblast (Ferro et al., 2008). Moreover, the adhesion of infected immune cells can also induce local destruction of the trophoblast (Juliano et al., 2006) through the induction of pro-inflammatory cytokine-dependent apoptosis (Garcia-Lloret et al., 2000; Juliano et al., 2006). It has also been proposed that the accumulation of leukocytes is responsible for placental villitis (Yavuz et al., 2006).

The ECM degradation is another strategy used by *T. gondii* to cross and damage the placental barrier (Wang and Lai, 2013; Liempi et al., 2020; Figure 3A.4). Placental infections increase the expression and secretion of MMPs, MMP-2, MMP-9, and MMP-12 in the VS, causing ECM degradation (Wang and Lai, 2013) particularly of fibronectin, collagen I and IV, and laminin (Chen and Aplin, 2003).

### Immune Response Against *Toxoplasma gondii*

*Toxoplasma gondii* infection in the placenta induces an inflammatory response characterized by a robust production of pro-inflammatory cytokines and chemokines (Sasai et al., 2018; Sasai and Yamamoto, 2019).

Toll-like receptors are critical in initiating defense against *T. gondii*; they recognize in the parasite glycosylphosphatidylinositol (GPI) anchored proteins and lipid anchors, heat shock protein 70 (TgHSP70), and profilin-like protein (TgPF) (Yarovinsky and Sher, 2006; Denkers, 2010; Yarovinsky, 2014; Sasai et al., 2018). GPI, are recognized primarily by TLR-2, TL-4 and TL2/TL1/6 heterodimers (Debierre-Grockiego et al., 2007) while TgPF is recognized by the TLR-5, 11 and -12 (Andrade et al., 2013; Salazar Gonzalez et al., 2014; Yarovinsky et al., 2005). On the other hand, parasite’s RNA and DNA are recognized by the endosomal TLR-7 and -9 (Yarovinsky, 2014). TLR activation leads to an increase of IL-12, IL-6, IL-8, and TNF-α cytokines, chemokines (CCL5, CCL12, and XCL1), interferons (IFNs), and other effector molecules such as NO, which favors infection control (Yarovinsky and Sher, 2006; Wujcicka et al., 2013).

Haplotype and single nucleotide polymorphisms (SNPs) of *tlr* genes can influence susceptibility to parasitic diseases (Wujcicka et al., 2013, 2017; Turkey et al., 2019). Thus, SNPs residing in TLR2, TLR4, and TLR9 genes increase susceptibility and development of toxoplasmosis in pregnancy (Wujcicka et al., 2013, 2014, 2017; Turkey et al., 2019). This susceptibility is attributed to the influence of SNPs in the receptor dimerization and the recruitment of adapter proteins involved in TLR signaling and the decreased synthesis of some cytokines (Wujcicka et al., 2017; Turkey et al., 2019), such as INF-γ and TNF-α.

We have shown previously that during *ex vivo* infection of human placental explants (HPE), *T. gondii* increases the expression of TLR-9, but not of TLR-2 and -4. However, inhibition of TLR-4 and -9 increases *T. gondii* DNA load in the explants. Notably, only IL-8 secretion was increased (Muñoz et al., 2016; Castillo et al., 2017a; Figure 3B); even the protective and pro-inflammatory cytokines INF-γ and TNF-α did not show a significant change in response to the parasite (Castillo et al., 2017a). This result could explain, at least partially, the susceptibility of the placenta to *T. gondii* infection compared to other parasites (Castillo et al., 2017a). In addition, it has been suggested that IL-8 promotes the proliferation and differentiation of the infected host cell, and subsequently, the intracellular multiplication of parasites (Milian et al., 2019), promoting, therefore, congenital transmission (Gómez-Chávez et al., 2019, 2020). Moreover, *T. gondii* Macrophage Migration Inhibitory Factor (TgMIF) cytokine-like, an analog to the host MIF, promotes IL-8 secretion and subsequent recruitment of immune cells to the site of infection (Sommerville et al., 2013) causing the above-described effects (Lambert et al., 2006).

On the other hand, the low INF-γ expression (Muñoz et al., 2016; Castillo et al., 2017a) could also be a placental mechanism to limit infection. Both INF-γ and TNF-α promote the adhesion of infected immune cells to the trophoblast through surface overexpression of ICAM-1 (Pfaff et al., 2005; Ferro et al., 2008). Therefore, it is possible that the downregulation of these pro-inflammatory cytokines, in addition to benefiting the maintenance of pregnancy, also limits the transfer of the parasite through the placental barrier. Furthermore, this could be related to the production of CCL22 chemoattractant in response to *T. gondii* (Ander et al., 2018) since CCL22 can replace the effect of IFN-γ and recruit Tregs modulating the inflammatory response. However, this increased T-reg recruitment may also be a parasite strategy to promote its persistence (Rowe et al., 2011). Furthermore, MIF is also overexpressed in responses to *T. gondii* infection (de Oliveira Gomes et al., 2011; Figure 3B), promoting the adhesion of infected immune cells to the trophoblast, activating the ERK1/2 MAPK pathway and subsequent production of prostaglandin E, favoring parasite proliferation and persistence through inhibition of IL-6, IL17, and TNF-α production (Kelly et al., 2005; Passos et al., 2010; Barbosa et al., 2014; Qiu et al., 2016).

The presence of *T. gondii* causes an imbalance of the Treg/T-helper type 17 (Th17) cells response inducing deleterious alterations in the placenta that can lead to abortion (Zhang et al., 2012). *T. gondii*, as mentioned above, promotes the production of IL-12, CCL-2, and CCL-22 activating naïve T cells. In addition, activated T cells secrete IL-2, INF-γ, and TNF-α, developing a pro-inflammatory environment that stimulates MΦ and B cells (Gómez-Chávez et al., 2020) and decreases Tregs and TGF-β levels (Figure 3B). The decrease of Tregs may be due to various factors, including apoptosis (Ge et al., 2008), limitation of proliferation (Oldenhove et al., 2009), and an increase in differentiation into Th17 cells (Zhang et al., 2012).

Importantly, all the factors involved in decreasing Tregs have in common the activation of multiple pathways, including the pro-inflammatory ones (Ge et al., 2008; Oldenhove et al., 2009; Zhang et al., 2012). The increased differentiation of TH17 cells is associated with altered expression of TGF-β, IL-17A, and IL-6 cytokines, transcription factors [i.e., Forkhead box-p3 (Foxp3)], and receptors [i.e., Retinoic acid-related orphan receptor γt (RORγt)], keys in cell differentiation and regulating Treg/Th17 balance (Zhang et al., 2012). Although it is unclear how *T. gondii* regulates the expression of TFG-B and Foxp3, it influences suppressive functions of Tregs in the placenta (Zhang et al., 2012), resulting in an exacerbated inflammatory reaction, causing placental damage and favors the transmission of the parasite to the fetus.

## Congenital Neosporosis

Congenital neosporosis affects mainly dogs and cattle, but occasionally also horses, sheep, and deer (Dubey, 2003), leading to abortion, stillbirth, and weak offspring (Mesquita et al., 2018). In sheep, congenital transmission rates are high, reaching values between 66.7 and 93% (González-Warleta et al., 2018). Transplacental transmission can be exogenous or endogenous. Exogenous transmission occurs following ingestion of sporulated oocysts by pregnant females, while endogenous one occurs as the reactivation of a pre-existent infection. The latter is the primary mechanism responsible for maintaining the parasite within livestock populations. The presence of the parasite induces an inflammatory response leading to placental damage and consequent abortion (Cantón et al., 2014b; Al-Shaeli et al., 2020).

**FIGURE 4:**
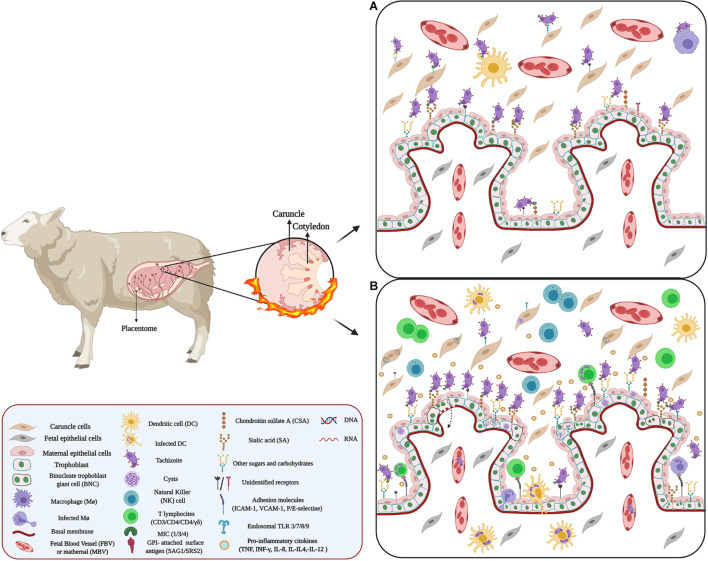
Host–pathogen interaction between *Neospora* and placenta: **(A)** Adhesion of Neospora. Neospora adhesion to the placenta can occur through proteins belonging to the SAG, SRS, and MIC families. These proteins expressed on the surface of the parasite act as surface antigens and ligands for molecules present on the surface of placental cells. Possibly, these protein proteins could bind to proteoglycans and placental adhesion molecules. **(B)** Microenvironment and inflammatory response against *Neospora*. Inflammation in the IVS and caruncle could be attributed to chemokines and cytokines secreted by maternal Mo, T cell, as well as ST and caruncle cells. Many of these cytokines and chemokines promote cell recruitment. Additionally, infection of some cells of the maternal immune system, such DCs, and MΦ, by *Neospora* alters the migration of these cells, using them as Trojan horses.

Thus, multiple necrotic lesions, thickening of the chorionic plate, and mineralization of necrotic foci can be observed in the infected ovine placenta. Notably, both the oocysts (sexual state) and tachyzoites (rapidly dividing parasite stage) of *N. caninum* can be detected in placental tissue (Mesquita et al., 2018; Al-Shaeli et al., 2020; Figure 4A). In the fetus, *N. caninum* causes severe damage to the brain, heart, lung, and liver (Al-Shaeli et al., 2020).

Although *N. caninum* is not considered a zoonotic parasite, serological studies indicate the possible capacity of *N. caninum* to infect humans, particularly immunocompromised individuals (Tranas et al., 1999; Lobato et al., 2006; Oshiro et al., 2015) and pregnant women. Recent reports revealed *N. caninum* in samples of umbilical cord blood and placental tissue in pregnant women. The seroprevalence in these women can vary between 8 and 24% (Ibrahim et al., 2009; Duarte et al., 2020) and is associated with the presence of domestic animals (Duarte et al., 2020). The biological similarities between *N. caninum* and *T. gondii* suggest the possibility of *N. caninum* transmission between humans (Petersen et al., 1999). Moreover, in studies where pregnant monkeys were inoculated with *N. caninum*, the transplacental transmission was evident; in the fetus, similar lesions as those caused by *T. gondii* can be observed (Barr et al., 1994; Ho et al., 1997). In mice, the congenital transmission of *N. caninum* occurs in 85% of the litters born from infected mothers (Cole et al., 1995).

### Adhesion of *N. caninum* to the Placenta

*Neospora caninum* can actively invade a large variety of nucleated cells *in vitro* and *in vivo* conditions (Naguleswaran et al., 2002; Hemphill et al., 2004; Lei et al., 2005); its mechanisms are very similar to those proposed for *T. gondii*, where a first stage of low-affinity adhesion and a second stage of firm apical attachment can be observed (Hemphill et al., 2004; Carruthers and Boothroyd, 2007).

The initial adhesion is mediated by tachyzoite surface antigens belonging to the SAG-related sequence proteins (SRS) family and homologous to the *T. gondii* SAGs family (Howe et al., 1998). SAG1 and SRS2 are two main immuno-dominant antigens of *N. caninum* tachyzoites (Dong et al., 2012; Sinnott et al., 2017) that binds to host cells (Hemphill et al., 2013). In addition, SRS allows parasite adhesion through binding to GAGs expressed on the host cell’s surface, especially CSA and SA (Dubey et al., 2017). The latter is crucial for self-recognition by complement factor H (Varki and Gagneux, 2012) and fetal defense against maternal complement attack (Abeln et al., 2019).

Then the apical attachment is mediated mainly by MIC (Lovett et al., 2000; Naguleswaran et al., 2002; Reid et al., 2012; Wang J. et al., 2017), MIC2 and MIC19 are unique to *N. caninum* (Reid et al., 2012). *N. caninum* MIC proteins have domains similar to those found in vertebrate ECM proteins, including thrombospondin (TPS) -like, integrin-like domain (Pereira et al., 2011) and SA- binding adhesive repeat- (MAR)-like domain (Friedrich et al., 2010). Thus, MICs can bind through GAGs to various receptors present on the surface of target cells (Dubey et al., 2017), and mediate cell adhesion (Keller et al., 2002; Wang J. et al., 2017). MIC1, MIC3, and MIC4 bind to host cells through sulfated proteoglycans (Naguleswaran et al., 2002; Keller et al., 2004; Friedrich et al., 2010). *N. caninum* proliferation varies between the different cells of the placenta (Gibney et al., 2008). Studies in bovine placenta cell lines have shown that caruncular cells appear to be more resistant to *Neospora* infection than trophoblasts due to their expression of adhesion molecules (Jiménez-Pelayo et al., 2017, 2019) and phagocytic activity (Machado et al., 2007; Jiménez-Pelayo et al., 2017). However, this resistance also depends on the virulence of the isolate (Jiménez-Pelayo et al., 2017). On the other hand, *N. caninum* shows a predilection for the fetal chorionic epithelium and placental blood vessels (Buxton et al., 1998).

There are few reports related to the adhesion molecules that participate in the placenta and *N. caninum* interaction. However, SRS2 participates in the adhesion of *N. caninum* to the trophoblast (Figure 4A) and the development of an effective immune response against transplacental transmission (Nishikawa et al., 2001; Haldorson et al., 2005, 2006), but the receptor for SRS2 has not yet been identified (Naguleswaran et al., 2002; Figure 4A). Nevertheless, the presence of SA and glycoproteins expressed on the surface of binucleate trophoblast giant cells (BNCs) in the ovine placenta (Jones et al., 1997; Klisch et al., 2006) could be involved in *N. caninum* adhesion to the placenta (Figure 4A).

Modulation of gene expression in the host cell is also a mechanism for *N. caninum* to invade and cross the placental barrier (Horcajo et al., 2017; Dorsch et al., 2019). Thus, infection of immortalized bovine trophoblasts with *N. caninum* induces the expression MMPs involved in ECM degradation (Cui et al., 2017; Horcajo et al., 2017). Additionally, infection by *N. caninum* alters the glycosylation pattern in the glycocalyx and apical cytoplasm of trophoblast (Dorsch et al., 2019; Figure 4A). The BNCs and the uterine epithelium also shows changes in sugars, including α-D-GalNAc, α-D-Man, β-D-Gal, α-D-Gluc, and NeuNac (Vonlaufen et al., 2004; Dorsch et al., 2019; Figure 4A). The expression of the adhesion molecules *E*-selectin, *P*-selectin, VCAM-1, and ICAM-1 is also altered, possibly through paracrine activation, through the pro-inflammatory cascade activation, which involves the release of IL-1b, IL-8, and MCP-1 leading to cell activation and subsequent expression of the adhesion molecules (Taubert et al., 2006).

*Neospora caninum* can also hijack immune cells to circumvent biological barriers. Thus, infection of DCs by tachyzoites enhances the translocation of parasites across cell monolayers (Collantes-Fernandez et al., 2012; Figure 4A). Furthermore, similar to *T. gondii* (Lambert et al., 2006; Lambert and Barragan, 2010), infection of DCs by *N. caninum* induce a migratory phenotype in these cells, enhancing its dissemination. In addition, *N. caninum* can infect MΦ and NK, cells and take advantage of their basal or inducible mobile properties (Boysen et al., 2006; Dion et al., 2011) (Figures 4A,B).

### Immune Response Against *Neospora*

The immune response at the maternal-fetal interface against *N. caninum* is characterized by a mixture of Th1 and Th2 responses, implying the presence of pro-and anti-inflammatory cytokines (Cantón et al., 2014b; Hecker et al., 2015; Arranz-Solís et al., 2016; Gutiérrez-Expósito et al., 2020; Figure 4B). Although these responses play a vital role in controlling parasite multiplication, they are responsible for placental damage that might lead to abortion (Maley et al., 2006; Rosbottom et al., 2011; Cantón et al., 2014a; Gutiérrez-Expósito et al., 2020).

The role of the innate immune response in the placenta against *N. caninum* has been studied mainly in cattle. The parasite is recognized by endosomal TLRs (3, 7, 8, and 9), which are upregulated in response to Neospora infection (Figure 4B; Marin et al., 2017).

Similar to *T. gondii* infection, TLR 3/7 and 8 is associated with the control of *N. caninum* intracellular infection. TLR7 activation leads to the development of a protective Th1 response (Andrade et al., 2013; Castillo et al., 2018) through MyD88-dependent signaling (Mineo et al., 2009; Miranda et al., 2019) inducing the secretion of pro-inflammatory cytokines including IFN-γ, IL-12, IL-18, and TNF-α, and promotes recruitment of T lymphocyte (CD3, CD4, CD8, and γδ) subsets, MΦ, NK cells (NKp46 subpopulation) (Maley et al., 2006; Cantón et al., 2014a, b; Hecker et al., 2015; Arranz-Solís et al., 2016; Gutiérrez-Expósito et al., 2020; Figure 4B).

In addition, the Th2 (IL-4) and regulatory (IL-10) cytokines also show alterations during *N. caninum* infection (López-Pérez et al., 2010; Rosbottom et al., 2011). Thus, the increase of IL-4 attenuates the effects of the pro-inflammatory response and enhances susceptibility to *N. caninum* favoring congenital transmission (López-Pérez et al., 2010). On the other hand, IL-10 has a regulatory effect on IFN-γ and TNF-α mediated response (Eperon et al., 1999; Quinn et al., 2004) and might also promote the invasion and intracellular replication of *N. caninum* and consequently its transmission.

## Conclusion

Apicomplexa are a large group of intracellular parasites that are distributed globally. Some of those parasites pose significant risks to pregnancy in humans and animals. The interaction between the parasites and hosts is the most critical factor in determining the success or failure of infection. During congenital infection, the parasites must break through the placental barrier and modulate host defenses. Apicomplexan parasites, despite having some differences during their life cycle, these parasites use very similar adhesion mechanisms to the placenta, based fundamentally on the alteration and modulation of the expression of molecules present on the surface of placental cells, including the trophoblast. Additionally, the presence of these parasites induces alterations in the immune response and a change in the Th1/Th2 balance, promoting the activation of defense mechanisms in the placenta based fundamentally on cellular immunity. This cellular immunity, mediated by different populations of T lymphocytes, secretion of chemokines, and pro-inflammatory cytokines, negatively affect the placental function and fetal growth that might ultimately cause fetal death and abortion.

Thus, better knowledge about changes in host gene expression, parasite strategies for modulating the host’s immune system, among others, should clarify the mechanisms of congenital transmission of those parasites.

## Author Contributions

MR-P prepared the draft of the manuscript the figures. LM, MR, AL, CC, EP-P, JG-M, and SA corrected the draft and figures and participated in the different rounds of correcting the manuscript. UK corrected the manuscript into its final version for submission.

## Conflict of Interest

The authors declare that the research was conducted in the absence of any commercial or financial relationships that could be construed as a potential conflict of interest.

## Publisher’s Note

All claims expressed in this article are solely those of the authors and do not necessarily represent those of their affiliated organizations, or those of the publisher, the editors and the reviewers. Any product that may be evaluated in this article, or claim that may be made by its manufacturer, is not guaranteed or endorsed by the publisher.
